# Variations in plant’s cry for help evidenced by modifications of rice root microbiota induced by blast or brown spot diseases

**DOI:** 10.1186/s40793-025-00787-2

**Published:** 2025-10-07

**Authors:** Léa Jobert, Gabriel Boulard, Nicolas Poncelet, Henri Adreit, Gilles Béna, Lionel Moulin

**Affiliations:** Plant Health Institute of Montpellier (PHIM), IRD, CIRAD, INRAE, Institut Agro, Univ Montpellier, Montpellier, France

**Keywords:** Oryza sativa, Phytopathogen, Pyricularia oryzae, Bipolaris oryzae, Microbiome assembly, Amplicon sequencing, Biocontrol

## Abstract

**Background:**

Plants can recruit specific microbes to help them defend themselves against phytopathogens in a process known as “cry for help”. In this study, we investigated whether a plant species modulates its root-associated microbiome differently - i.e. “cries out differently” - depending on the diversity of fungal pathogens attacking its leaves. To address this question, we monitored changes in the root microbiome of *Oryza sativa* cv. Nipponbare following infection with two fungal pathogens: *Pyricularia oryzae* (leaf blast) and *Bipolaris oryzae* (brown spot), under controlled conditions and using the same soil.

**Results:**

Our results support the “cry for help” hypothesis, suggesting that pathogen-induced stress drives the recruitment of beneficial microbes. While the composition of the root-associated microbiota remained globally stable after infection, subtle but significant taxonomic shifts were observed. Alpha diversity was unaffected, but changes in beta diversity occurred in micro-eukaryotic communities one week after brown spot infection and in bacterial communities two weeks after blast infection. Notably, beneficial taxa such as the bacterial genera *Lentzea* and *Streptomyces*, as well as the fungi *Cladosporium halotolerans* and *Rhizophagus irregularis*, were enriched in the below-ground microbiome of leaf-infected plants. The biocontrol potential of *Rhizophagus irregularis* was confirmed against blast but not brown spot infection.

**Conclusions:**

These results advance our understanding of the “cry for help” hypothesis in rice and provide potential candidates for biocontrol. They highlight the complexity of plant-microbe interactions and suggest that rice plants specifically modulate their root microbiome in response to fungal infections, potentially shaping microbial communities to enhance defence strategies.

**Supplementary Information:**

The online version contains supplementary material available at 10.1186/s40793-025-00787-2.

## Background

Plants are under constant pressure to defend themselves against a variety of biotic and abiotic stresses and are therefore adopting various strategies to withstand them. In addition to internal immunological strategies, plants have been shown to employ external strategies that rely on above- and belowground recruitment of beneficial insects and microorganisms [1]. A number of studies have shown that plants under long-term stress from soilborne pathogen can enrich antagonistic microorganisms to create a disease-suppressive soil, thereby reducing the severity of soilborne diseases [2–4]. Disease-suppressive soils typically develop after prolonged disease outbreaks, suggesting that plants may assemble a protective microbial community in response to pathogen infection [5]. The “cry for help” hypothesis provides a mechanistic explanation for the development of disease-suppressive soils. This hypothesis proposes that plants recruit beneficial microbiota, through the release of stress-induced exudates, to resist various stresses they encounter [2, 6]. The “cry for help” can be modelled into four successive stages: (I) plants are attacked by pathogens or herbivores, which trigger local and systemic signals that activate root immunity; (II) changes in root exudation profiles occur leading to (III) recruitment of beneficial root microbes that alter root microbiome activities and in the final stage, (IV) this altered microbiome antagonises pathogens via direct or indirect mechanisms [7]. Several studies have shown the build-up of beneficial microbial communities in the roots mediated by changes in root exudation following a foliar infection. Rudrappa et al.. showed that infection of *Arabidopsis thaliana* leaves with *Pseudomonas syringae* pv. *tomato* (*Pst*) induced an increased root exudation of malic acid which favored the recruitment of a beneficial *Bacillus subtilis* triggering Induced Systemic Resistance (ISR) [8]. Foliar infection of *Arabidopsis thaliana* by *Hyaloperonospora arabidopsidis* (*Hpa*) specifically promoted three bacterial species in the rhizosphere that together stimulate plant growth and the induction of plant defences against *Hpa* [5]. Many plant growth-promoting bacteria (PGPB) are well-documented for their ability to trigger ISR in plants, thereby enhancing their defence mechanisms [9]. Similarly, arbuscular mycorrhizal fungi (AMF) can boost plant immunity through a related process known as mycorrhiza-induced resistance (MIR) [10].

The hypothesis that plants can actively recruit a beneficial microbiota as a defence mechanism not only supports our understanding of disease-suppressive soils but also emphasizes the dynamic nature of plant-microbe interactions [3, 11]. Despite its potential importance, research into the “cry for help” hypothesis remains limited and focused on the model plant *Arabidopsis*, so further investigation is needed to fully understand the mechanisms involved and their implications for sustainable agriculture.

In our study, we focused on rice (*Oryza sativa* L.), a crucial global staple feeding over 4 billion people worldwide. Given the steady growth of the global population, the demand for this crop is expected to keep increasing [12]. However, pathogens and pests are estimated to have reduced global rice production by around 30%, making it the most affected of the world’s five major food crops [13]. The two most devastating foliar diseases affecting rice production worldwide are blast and brown spot, caused by the fungi *Pyricularia oryzae* and *Bipolaris oryzae*, respectively [13, 14]. These fungal diseases have a significant economic impact, with yield losses ranging from 10 to 30% for rice blast [15] and up to 52% for brown spot [16]. In rice, ISR has been effectively induced by strains of *Pseudomonas fluorescens* and *Bacillus subtilis* to combat blast disease, while AMF have also been shown to enhance rice resistance to *Pyricularia oryzae* through the activation of MIR pathways, with variations of this response depending on the rice cultivar and AMF species [17, 18].

To our knowledge, only a few studies have investigated the effect of pathogen infection of rice leaves on root microbiota: one induced by bacterial leaf blight and another by blast infection [19–22]. Bacterial Leaf Blight (BLB) infection significantly affected the microbial community composition and certain genera were statistically enriched or depleted in diseased samples [19]. Following blast infection, the alpha diversity of root-associated fungi or bacteria didn’t exhibit any changes, nor did the beta diversity [22] but a substantial alteration of bacterial community structure was however observed in root and rhizosphere compartments in another study [21]. These studies all looked at more subtle changes and found significant enrichment or depletion in the abundance of specific fungal or bacterial genera following foliar infection. Still no studies have compared the modification of the rice microbiota to infection by different phytopathogens to unravel common and specific responses. Thus, it is still unknown whether the recruitment of beneficial microorganisms by plants during foliar infection is disease specific.

In this study, our main objective was to investigate the variation in the “cry for help” of rice (using root microbiome variations as a proxy) under attack by two different leaf fungal phytopathogens. We worked with soil sampled from a rice field in Camargue region (France) where these two diseases have been previously documented [23].

We first described the rice root-associated microbiome from the rice field under investigation, then inferred how this microbiome changes after infection of *Oryza sativa* subsp. *japonica* cv. Nipponbare with two fungal foliar pathogens: *Pyricularia oryzae* and *Bipolaris oryzae*. The root-associated microbiota from healthy and diseased plants, was investigated using metabarcoding amplicon sequencing (bacteria (16 S rRNA gene V3V4) and micro-eukaryotes (ITS2), and a “cry for help”-responsive fungal taxa, the AMF *Rhizophagus irregularis*, was further investigated for its bioprotective capacity on rice infected by *B. oryzae* or *P. oryzae*.

## Methods

### Soil and pots preparation

Soil was collected from Gageron in the Camargue (France) (43,625 N, 4,606E) at a depth of 0–15 cm on 28th of February 2023 and stored in bags at room temperature for one week, before further treatment. The field from which the soil was taken was under conservation agriculture management with the rice variety “Arélate”. At the time of sampling, the rice season had been over for 4.5 months and rape and alfalfa were grown as cover crops.

The soil was first sorted to remove larger root fragments, stones, earthworms, and other insects. The soil was then lightly air-dried and fragmented into smaller pieces to facilitate sieving. The Camargue soil was mixed with 80% sterilized potting soil and 1 g/L of fertilizer (LDTwin Plus 19-5-8, Compo Expert) using a concrete mixer. The potting soil had previously been autoclaved twice at 5-day intervals under wet cycle conditions. The mixture was stored in ventilated containers for 10 days to stabilize the microbiota. After the resting period, the soil was remixed using the concrete mixer and then distributed into 650 ml anti-coiling pots (Comptoir Vert, France) with autoclaved filter paper placed at the bottom to prevent root protrusion and cross-contamination through pot drainage holes.

### Plant growth

*Oryza sativa* L. subsp. *japonica* cv. Nipponbare was used as the plant model because of its sensitivity to both diseases and because the pathosystems of this cultivar are mastered in our laboratory. The seeds, which have been maintained and propagated in our laboratory for several years, were originally obtained from the International Rice Research Institute (IRRI). Prior to the experiment, seeds were dehusked using the automatic rice husker TR-260 (Kett Electric Laboratory) and surface-sterilized. Briefly, seeds were immersed in 70% ethanol for 3 min, followed by three rinses with sterile distilled water. Subsequently, the seeds were immersed in sterile distilled water at 28 °C for 24 h and then transferred to petri dishes containing sterilized sand for pregermination in a culture chamber [24]. After 48 h, homogeneously germinated seeds were selected and transplanted into pots, with three seedlings per pot to ensure uniformity in plant growth. Within 48 h post-sowing, only one seedling per pot was retained for further experiments. Plants were cultivated for three weeks in a growth chamber with a photoperiod of 12 h day/night, maintained at 28 °C day and 26 °C night, and a relative humidity of 75%.

### Inoculation and sampling

After three weeks of growth, the plants were randomly divided into four groups of 10 plants each: one group inoculated with *Pyricularia oryzae* (syn. *Magnaporthe oryzae*) and its respective control, and another group inoculated with *Bipolaris oryzae* along with its corresponding control. *P. oryzae* FR13 was isolated from *Oryza sativa* in Camargue, France, in 1988 [25], while *B. oryzae* FR9037 was isolated on rice in Camargue in the south of France (2018) [26], both being kept in the laboratory. Inoculum preparation and inoculation were conducted as described in [27]. Briefly, isolates were grown on rice flour for 11 days for *Pyricularia* or on potato dextrose agar (PDA) for 9 days for *Bipolaris*. Spores were collected by scraping and rinsing plates filled with distilled water. After filtering through two layers of nylon mesh, spores were counted using a Malassez counting chamber. Finally, inoculum was prepared at a concentration of 70,000 spores/ml (*Pyricularia*) or 20,000 spores/ml (*Bipolaris*) in pure water containing 0.5% gelatin.

Plants were inoculated by spraying each plant from each side, so that all the leaves were covered with inoculum. Control plants were sprayed with 0.5% gelatin solution (mock treatment). All plants were placed overnight in a transparent plastic box with a humidifier overnight, to increase humidity to 100%. The plants were kept under the plastic box until the end of the experiments, but the box was opened after 12 h. Sampling was timed to coincide with the development of symptoms: plants inoculated with *Bipolaris* and corresponding controls were sampled one week after inoculation, while *Pyricularia* and corresponding controls were sampled after two weeks. Roots with adherent soil were frozen in liquid nitrogen and stored at -80 °C.

### DNA extraction and amplicon-barcoding data production

Rice roots with adherent rhizosphere were ground in a sterile mortar using liquid nitrogen and 250 mg of powder was transferred to PowerBead tubes (Qiagen). Total DNA was extracted using the DNeasy PowerSoil Pro Kit (Qiagen) according to the manufacturer’s instructions. Negative controls for extraction kit were included. DNA samples were stored at -20°C until further use. DNA quality control, PCR amplification, library construction and MiSeq Illumina sequencing were performed by Macrogen (Seoul, South Korea) using 337F (5’-CCTACGGGNGGCWGCAG-3’) and 805R (5’-GACTACHVGGGTATCTAATCC-3’) primers to amplify the V3-V4 region of the 16 S rRNA gene [28] and ITS-3 F (5’-GCATCGATGAAGAACGCAGC-3’) and ITS-4R (5’-TCCTCCGCTTATTGATATGC-3’) to amplify the ITS2 region of the rDNA Internal Transcribed Spacer (ITS) [29]. The sequencing libraries were prepared according to the Illumina 16 S and ITS2 Metagenomic Sequencing Library protocols. The input gDNA (5 ng for 16 S, 10 ng for ITS2) was PCR amplified with 5x reaction buffer, 1 mM of dNTP mix, 500 nM each of the universal F/R PCR primer, and Herculase II fusion DNA polymerase (Agilent Technologies, Santa Clara, CA). The cycle condition for first PCR was 3 min at 95 °C for heat activation, and 25 cycles of 30 s at 95 °C, 30 s at 55 °C and 30 s at 72 °C, followed by a 5-min final extension at 72 °C. The first PCR product was purified with AMPure beads (Agencourt Bioscience, Beverly, MA). Following purification, 2 µl of first PCR product was PCR amplified for final library construction containing the index using Nextera XT Indexed Primer. The cycle condition for second PCR was same as the first PCR condition except for 10 cycles. Positive (known gDNA) and negative controls (no gDNA) were added for each PCR to assess performance and contamination, respectively. The PCR products were purified with AMPure beads. The final purified product was quantified using qPCR according to the qPCR Quantification Protocol Guide (KAPA Library Quantification kits for Illumina Sequencing platforms) and qualified using the TapeStation D1000 ScreenTape (Agilent Technologies, Waldbronn, Germany). The paired-end (2 × 300 bp) sequencing was performed by the Macrogen using the MiSeq™ platform (Illumina, San Diego, USA). The number of sequences varied from 17,900 to 29,700 (mean at 24,400) for 16 S rRNA gene libraries and from 25,200 to 54,900 (mean at 37,600) for ITS libraries. The amplicon sequencing data (fastq) for this study are accessible in the ENA (European Nucleotide Archive, https://www.ebi.ac.uk/ena) database under the Bioproject PRJEB84129.

### Bioinformatic analysis of ITS and 16 S rRNA gene sequences

After performing a quality screening, raw sequences were processed into exact sequence variants (ESVs) using DADA2 [30]. The DADA2 algorithm is resolving amplicon sequence variants (ASVs) that differ by as little as one nucleotide [30]. Briefly, the DADA2 R-package was used to perform quality filtering, dereplication, error rate learning, sample inference, chimera identification, merging of paired-end reads and taxonomic classification. Taxonomic assignments were made using the SILVA v138 database [31] for 16 S rRNA gene sequences and the UNITE (sh_general_release_dynamic_s_all_25.07.2023_dev) for ITS2 sequences. ASVs that were not affiliated at the Kingdom or Phylum level were excluded, as were ASVs affiliated to chloroplasts, mitochondria (16 S rRNA gene dataset) and *Viridiplantae* at Kingdom level (ITS dataset). Rarefaction curves and alpha diversity figures were generated using the phyloseq and ggplot2 packages in R studio. NAMCO, an R shiny application for microbiome analysis was used for downstream microbiota diversity analyses [32]. After uploading metadata and dataset, filtering was performed to remove ASVs with < 10 reads across all libraries, and data were normalised using center-log ratio method. The final dataset used for taxonomic binning contained 1901 ASVs for 16 S rRNA and 704 ASVs for ITS. Analysis of rRNA and ITS sequences were conducted primarily at the genus level as species-level identification was also not always achievable. Beta diversity analyses (non-metric multidimensional scaling - NMDS, permutational multivariate analysis of variance – PERMANOVA (with 999 permutations) and size effects) were performed using Phyloseq and Vegan. A Wilcoxon test (threshold *p-value = 0.05*) was performed to compare enriched or depleted taxa between inoculated and control samples using ALDEx2 package. The boxplots for relative abundance of individual taxa were generated using the packages “ggplot2” and “dplyr” in Rstudio. The relative abundances of normalised data were visualized using “heat trees”, created with the “metacoder” package [33]. In these trees, the size of nodes and edges are correlated with the abundance of organisms in each community and colour of nodes and edges are correlated with differential abundance between conditions (log2foldchanges), respectively. R scripts for the DADA2 pipeline, rarefaction curves, alpha diversity and metacoder heat trees are available on github at https://github.com/lmoulin34/Lea_PyriBlast.

### Biocontrol test using Rhizophagus irregularis

*Rhizophagus irregularis* (strain DAOM 197198) was obtained from MycAgro Lab (Bretenière, France) as a granular inoculum containing 100 spores per gram. Four conditions were tested: inoculated or not with *Rhizophagus irregularis;* and leaf-infected with *Bipolaris oryzae* or *Pyricularia oryzae*. A total of 20 plants per condition were grown in anti-coiling pots (Comptoir Vert, France) with a layered substrate consisting of 150 mL of clay beads (6–18 mm, Terres & Traditions, France) at the bottom and 450 mL of sterilized substrate. The substrate was composed of 40% potting soil, 25% sand, 20% perlite, and 15% vermiculite. After autoclaving, the substrate was inoculated with either the arbuscular mycorrhizal fungi (AMF) granular inoculum or a control inoculum without fungal spores, at a proportion of 5% (v/v) per pot. The *Oryza sativa L.* subsp. *japonica* cv. Nipponbare seeds used in the study were surface sterilized and germinated as described above. Plants were watered three times a week. From the third week, they were supplied with a modified Yoshida nutrient solution [34], depleted of phosphate to promote mycorrhization.

Rice plants were grown for two months prior to pathogen infection. Pathogen inoculations were carried out as described in the Methods section. Sampling was done five days post-infection for brown spot and eleven days post-infection for blast, a timing chosen according to symptoms development for each disease. The last three leaves of each plant were scored using a standardized disease severity scale (0–9) from [35]. Disease severity was determined by using the formula from [36]: “Disease severity (%) = Sum of total scores / (Total number of observations × highest score in the scale) × 100”. As the data didn’t follow a normal distribution (Shapiro test, *p-value < 0.05*), a non-parametric Wilcoxon test was performed to assess statistical differences between conditions.

To assess mycorrhization, root systems from five plants per condition were sampled. After removing soil residues, roots were washed with tap water, immersed in 70% ethanol, and stored at 4 °C until further processing. Fungal structures were visualized using a blue ink staining protocol adapted from [37]. Roots were cleared in 10% KOH at 80 °C for 45 min, rinsed five times with ultrapure water (MilliQ), and stained with a solution of 5% blue ink (Waterman “Bleu Sérénité”) in 5% acetic acid for 10 min at room temperature. Stained roots were washed five more times with ultrapure water and stored overnight in 5% acetic acid. Ten fragments of coronary roots per plant were mounted and examined under an Axiozoom Zeiss microscope to verify the presence of mycorrhization.

## Results

### Capturing rice root-associated microbial diversity from Camargue soil

**Fig. 1:**
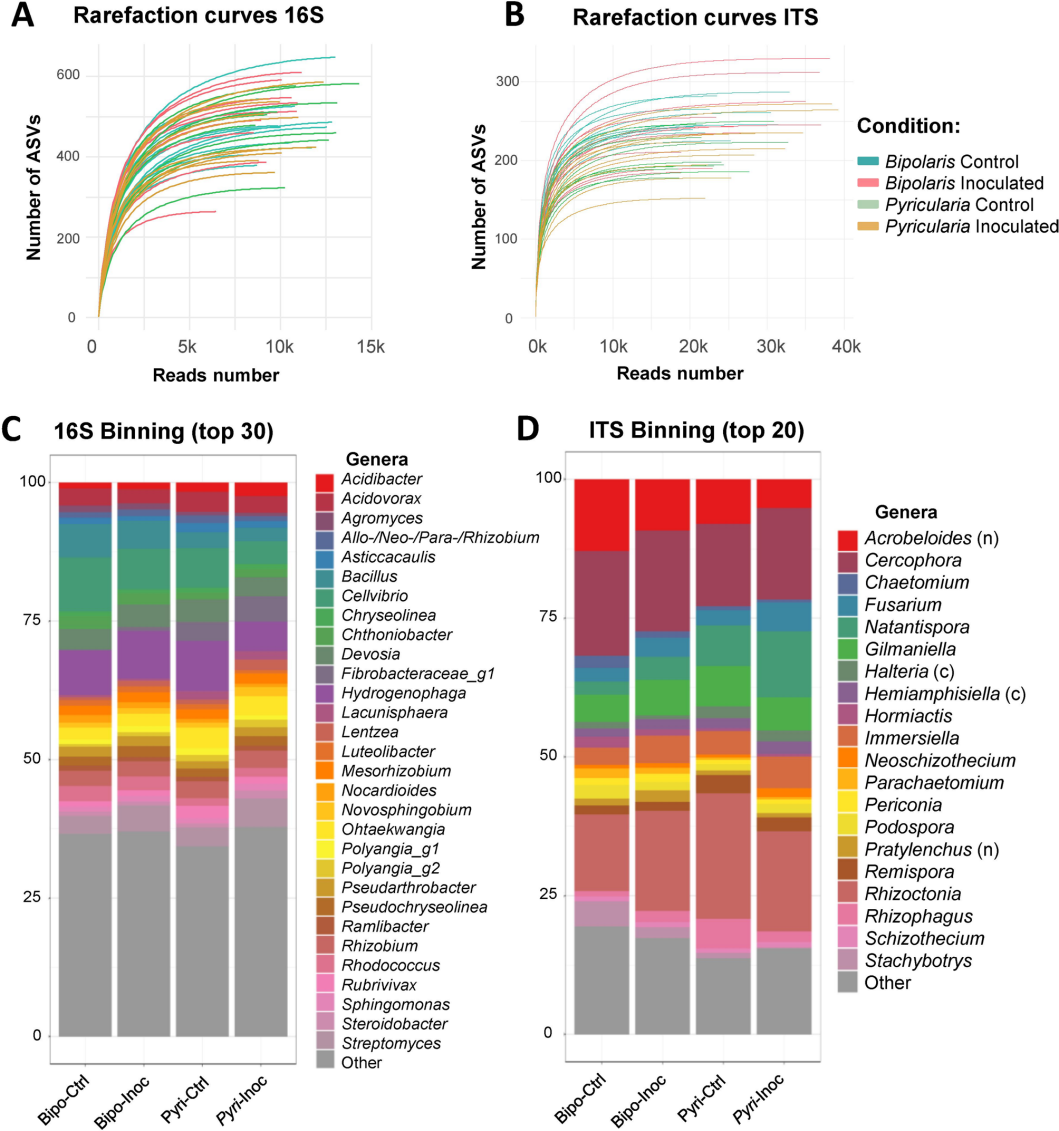
Rarefaction curves (**A**, **B**) and taxonomic binning (**C**, **D**) of 16 S and ITS ASVs. Taxonomic binning was made on relative abundance of genera, on filtered and normalised data (center-log ratio), at genus level for the top 30 most abundant 16 S (B) and top 20 most abundant ITS (D) in the root-associated microbiota. Abbreviations: Bipo-Ctrl and Pyri-Ctrl: uninoculated controls used for each pathosystem; Bipo-Inoc and Pyri-Inoc: inoculated conditions for each fungus (Bipo: *Bipolaris oryzae*, Pyri: *Pyricularia oryzae*); (n) Nematoda, (c) Ciliophora

*Oryza sativa* cv Nipponbare plants were grown on a soil from Camargue and inoculated with either *Bipolaris oryzae* or *Pyricularia oryzae* and their rice-root-associated microbiome diversity was analyzed using 16 S rRNA amplicon gene for bacteria and ITS for microeukaryota. Reads quality and filtering steps are given in the Material and Methods section. The depth of amplicon sequencing was sufficient to capture the diversity of bacteria and microeukaryotes associated with the rice root, as shown by the rarefaction curves (Fig. 1A and B).

The top 30 most abundant bacterial genera present in all samples are shown in Fig. 1C, with the most commonly detected bacterial genera being *Bacillus*, *Cellvibrio*, *Devosia*, *Hydrogenophaga* and *Streptomyces*.

In contrast to 16 S rRNA gene sequences, where the top 5 sequences account for only about 25% of the relative abundance, the top 5 ITS sequences account for more than 50%. The top 20 ITS genera are shown in Fig. 1D and are dominated by fungi, with the exception of two nematodes and two ciliates. The most frequently detected fungi belong to the genera *Cercophora*, *Rhizoctonia*, *Gilmaniella* and *Natantispora*. Additionally, among the five most abundant ITS sequences, we detected bacterivorous nematodes belonging to the genus *Acrobeloides*.

### Blast and brown spot impacts on alpha and beta diversities of rice root associated microbiota

**Fig. 2:**
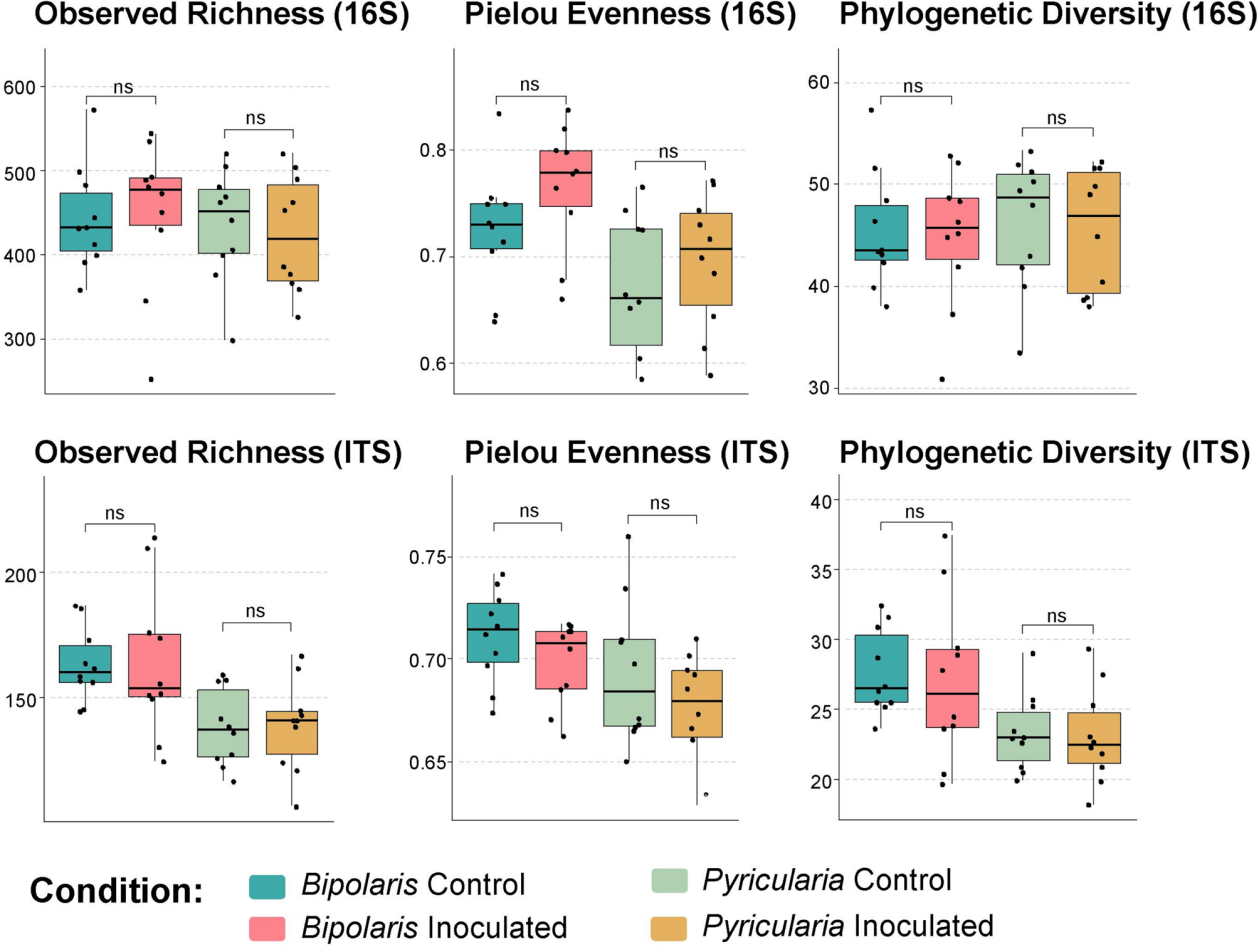
Comparison of ASV alpha diversity in the root-associated microbiome of control and inoculated plants with *Bipolaris oryzae* or *Pyricularia oryzae*. Wilcoxon statistical tests were performed between treatments (ns: non-significative at alpha = 5%)

Alpha diversity using species richness, Pielou evenness or phylogenetic diversity was measured for both bacterial and fungal communities as shown in Fig. 2. No significant differences in bacterial and fungal alpha diversity indices were observed between the control and infected samples for any of the pathosystems tested (*p > 0.05*).

**Fig. 3:**
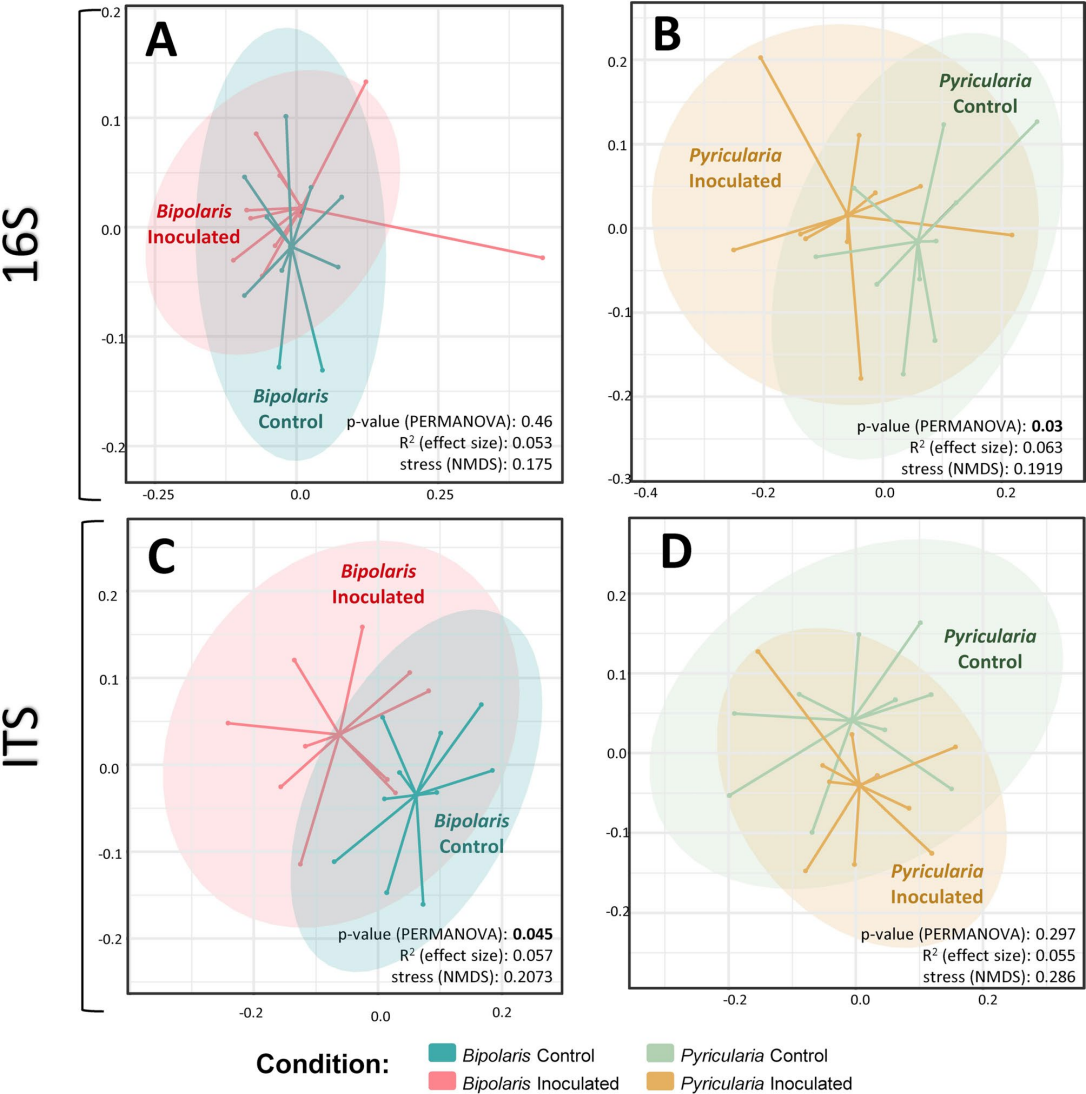
NMDS ordination showing the comparison in the structure of the root-associated microbiome. NMDS of control and *Bipolaris*-inoculated plants (16 S (**A**) and ITS (**C**)) and control and *Pyricularia*-inoculated plants (16 S (**B**) and ITS (**D**)). *P*-values of PERMANOVA test (inoculated versus not inoculated), R^2^ effect size and NMDS stress and are indicated on the bottom left side of each NMDS

To compare the microbial community structures between the different treatments, NMDS analyses on 16 S and ITS sequences were performed using Bray-Curtis dissimilarity and are presented in Fig. 3. PERMANOVA tests were performed to compare inoculated from control plants for either *Bipolaris* or *Pyricularia* pathosystems. An important variability among the biological samples was observed but significant differences were detected for *Bipolaris* inoculated versus *Bipolaris* control ITS sequences (*p = 0.045*). This change in the beta diversity of rice root associated microeukaryota upon infection by *Bipolaris* is not observed for bacteria. In the case of *Pyricularia* infection, we observed a significant difference in the beta-diversity of bacterial communities between infected and non-infected samples (*p = 0.*03), while no significant differences could be detected for the beta diversity of microeukaryotes (Fig. 3), highlighting the different results on changes in the root microbiota for each pathogen.

### Blast and brown spot affect taxonomic composition in rhizosphere communities

**Fig. 4:**
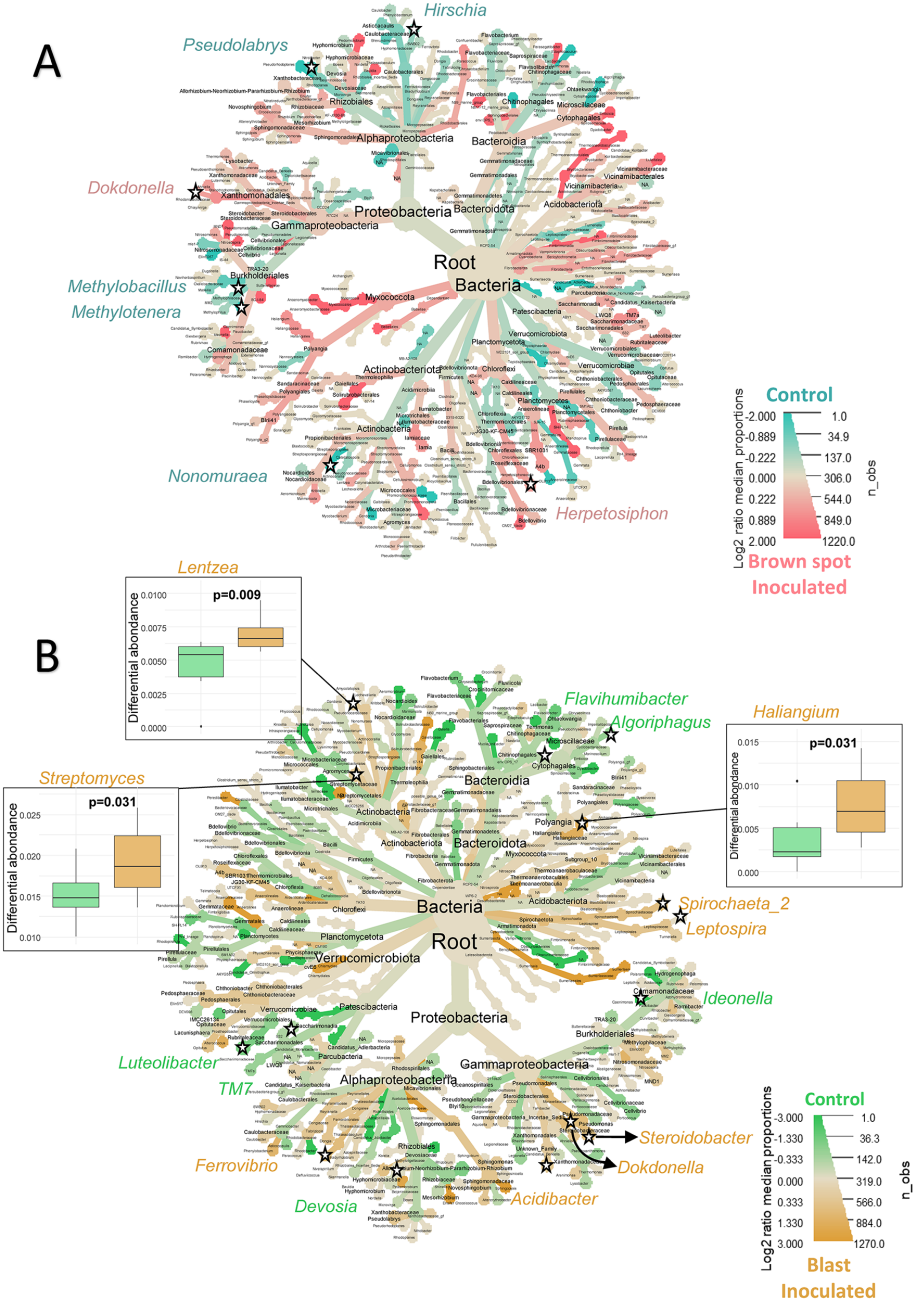
Heat trees of enrichment or depletion of taxa in the 16 S dataset between control and leaf-inoculated plants with *Bipolaris oryzae* (brown spot) (**A**) or *Pyricularia oryzae* (blast) (**B**). The colour scale corresponds to log2-fold enrichment, with pink or orange indicating enrichment in inoculated samples and blue or green indicating depletion in inoculated samples. Significant Wilcoxon test (α = 0.05) on centre-log ratio normalised data of control and inoculated plants are indicated by an asterisk. Several box plots of statistically different abundant taxa are shown around the tree; all box plots of statistically different abundant taxa are available in the Supplementary Material S1 and S2

**Fig. 5:**
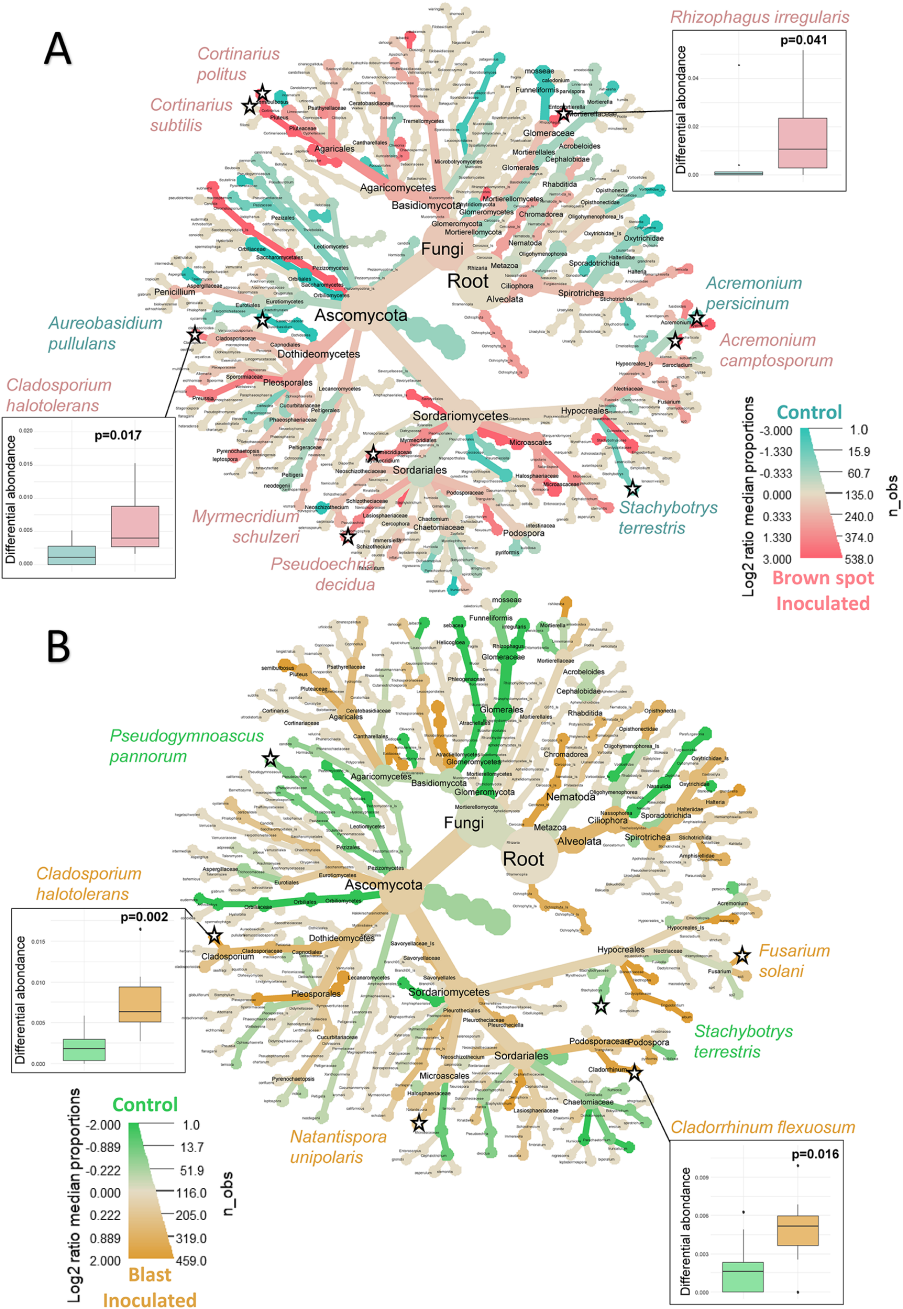
Heat trees of enrichment or depletion of taxa in the ITS dataset between control and leaf-inoculated plants with *Bipolaris oryzae* (brown spot) (**A**) or *Pyricularia oryzae* (blast) (**B**). The colour scale corresponds to log2-fold enrichment in inoculated samples compared to controls. Significant Wilcoxon test (α *= 0.05*) on centre-log ratio normalised data of control and inoculated plants are indicated by an asterisk. Several box plots of statistically different abundant taxa are shown around the tree; all box plots of statistically different abundant taxa are available in the Supplementary Material S3 and S4

For each pathosystem, the differential enrichment of rice-root associated microbial taxa between inoculated and control plants was analyzed. Using Metacoder, the log2 fold differential relative abundance of each ASV between inoculated and control samples was visualized in a heat tree for 16 S rRNA gene sequences (Fig. 4A and B) and ITS sequences (Fig. 5A and B).

By examining each taxon in detail, many showed significant relative abundance differences between inoculated and healthy plants in both bacterial and microeukaryotes composition for both diseases.

In the root-associated bacteria inoculated with *Bipolaris*, two identified genera were enriched when compared with healthy samples: *Dokdonella* (+ 70%), and *Herpetosiphon* (+ 108%), and five were depleted: *Hirschia*, *Pseudolabrys*, *Methylobacillus*,* Methylotenera* and *Nonomuraea* (Fig. 4A, detailed analysis in Supplementary Material S1). In the case of *Pyricularia* infection, nine genera were more abundant in diseased plants with an increase ranging from 20% to 283% (Fig. 4B, Supplementary Material S2): *Lentzea*,* Streptomyces*, *Ferrovibrio*, *Acidibacter*, *Dokdonella*, *Steroidobacter*, *Leptospira*, *Spirochaeta_2* and *Haliangium*, whereas six genera were statistically enriched in healthy plants (+ 20% to + 100%): *Luteolibacter*, *TM7*, *Devosia*, *Ideonella*, *Algoriphagus* and *Flavihumibacter*.

For the ITS marker, *Bipolaris* infected plants showed a statistically significant enrichment of *Rhizophagus irregularis* (AMF), *Cortinarius politus*, *Cortinarius subtilis*, *Cladosporium halotolerans*, *Myrmecridium schulzeri*, *Pseudoechria decidua* and *Acremonium camposporum* with increases between 186% and 1756% corresponding to an 18.5-fold increase (Fig. 5A, Supplementary Material S3). Brown spot uninfected plants were enriched in *Aureobasidium pullulans*, *Stachybotrys terrestris* and *Acremonium persicinum*. For *Pyricularia*-infected plants, less fungal species were found differentially abundant between healthy and diseased plants: *Cladosporium halotolerans*, *Natantispora unipolaris*, *Cladorrhinum flexuosum* and *Fusarium solani* were enriched in diseased plants whereas *Pseudogymnoascus pannorum* and *Stachybotrys terrestris* were depleted in inoculated plants (Fig. 5B, Supplementary Material S4).

### Rhizophagus irregularis inoculation on rice roots reduce symptoms of blast disease but not brown spot under greenhouse conditions

**Fig. 6:**
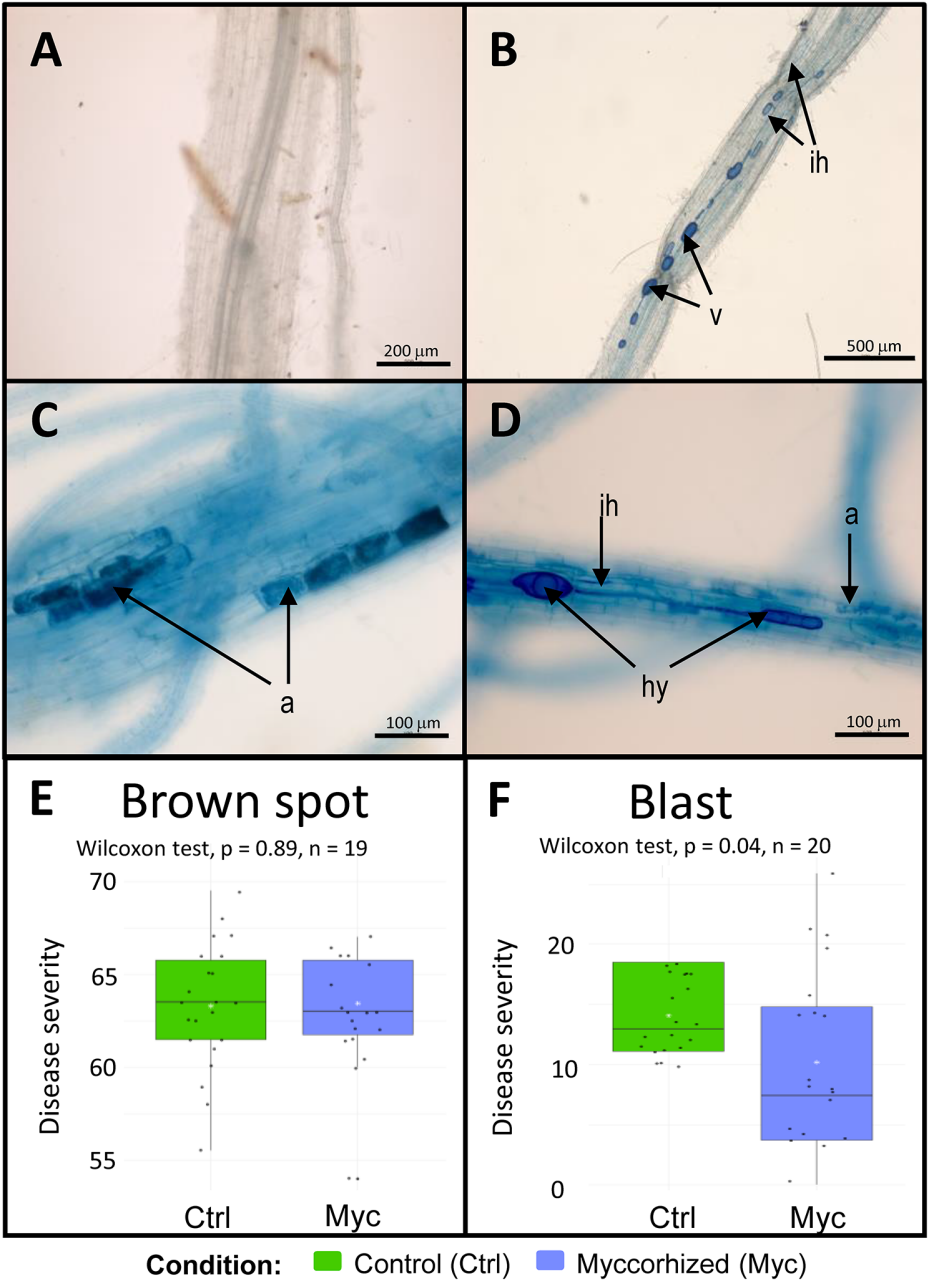
Rhizophagus irregularis (AMF) colonisation of Nipponbare roots (**A**-**D**) and its impact on brown spot and blast disease symptoms (**E**-**F**). **A**: Nipponbare roots of control plants without AMF; **B**: roots colonized by *R. irregularis* showing intercellular hyphae (ih) and vesicles (v); **C**: colonised roots with arbuscules (a), **D**: colonized roots with intercellular hyphae (ih), hyphopodia (hy) and arbuscules (a); **E**: impact of root mycorrhization on brown spot disease severity; **F**: impact of root mycorrhization on blast disease severity. *n* = number of plants

The AMF *Rhizophagus* (*R.*) *irregularis* was identified as enriched in the root-associated microbiome of brown spot-infected plants (Fig. 5A). As this species is available for commercial purchase, we evaluated its potential biocontrol activity against blast and brown spot diseases. Two-month-old rice plants, either inoculated with *R. irregularis* or with a spore-free control inoculum, were infected with *B. oryzae* or *P. oryzae*. AMF root colonization was observed in roots inoculated with *R. irregularis* and not in uninoculated controls (Fig. 6A). Symbiotic AMF organs such as arbuscules were observed on mycorrhized plants (Fig. 6B and D). Plants were harvested five days after brown spot infection and seven days after blast infection and disease severity was measured as described in Material and methods. Mycorrhization had no effect on brown spot severity, whereas for blast, mycorrhization reduced disease severity by 27% (Wilcoxon test, *p* = 0.04) (Fig. 6E-F).

## Discussion

Plants face constant biotic and abiotic stresses, and defend themselves through both internal immunity and the recruitment of beneficial microbes above- and below-ground [1]. As holobionts, they shape their microbial communities upon infection for self-protection against subsequent multiple attacks, a process known as “crying for help” [38]. Here, we investigate whether a plant modulates its root-associated microbiome differently depending on the diversity of foliar pathogens attacking it.

### Rice-root associated microbial diversity from Camargue soil

The Camargue region is a 750 km² delta in southern France, favorable for rice production but with high salinity [39, 40]. Little is known about bacterial diversity in the rice rhizosphere under these conditions, so we first examined bacterial and micro-eukaryote communities, focusing on the most abundant genera.

Among the top 30 bacterial genera, many are previously described in rice (e.g., *Sphingomonas*, *Rhizobium*, *Acidovorax*, *Bacillus*) [41, 42], while some commonly found core microbiome rice genera such as *Herbaspirillum*, *Pantoea*, *Pseudomonas*, *Xanthomonas* or *Microbacterium* were surprisingly not found in high abundance in our study [41, 42]. This result may be attributed to the fact that at the time of sampling, the rice season had ended 4.5 months earlier, and rapeseed and alfalfa were being cultivated in the field, likely influencing the local soil community, or that these genera are not adapted to the particular Camargue soil condition (high salinity).

Compared to previous studies in Camargue [39], most abundant genera differ, except for *Sphingomonas* and the halophilic genus *Haliangium*. This result is not particularly surprising as we sampled in the same region but not at the same site, ten years later and not at the same time of year.

Among our top 30 bacterial genera, only the genus *Acidovorax* is known to contain a rice phytopathogenic species causing bacterial brown stripe on rice: *Acidovorax avenae* subsp. *avenae* [43]. The genus *Sphingomonas* is known to contain species with growth promoting effects on rice (as *Sphingomonas panacis*) [44] but a potential pathogenic species causing bacterial leaf blight has been reported in African countries [45]. We detected several bacterial genera known to contain potential rice growth promoting species such as *Bacillus*, *Devosia*, *Rhizobium* and *Streptomyces* [46–51]. *Cellvibrio* strains are frequently isolated form soil and rhizosphere, and some species car perform cellulose degradation, suggesting a potential role in the decomposition of organic matter within the rice rhizosphere [52]. The presence of these genera highlights the diversity of functional groups of bacteria associated with rice, ranging from nutrient cycling to potential crop protection.

Among the top 20 genera in the ITS dataset, the majority are fungi, but there are also two nematodes: *Acrobeloides*, which is a bactivorous genus, and *Pratylenchus*, with some species known to cause root lesions in rice [53] and two ciliates genera: *Halteria* and *Hemiamphisiella*. The genera *Fusarium*,* Cercophora* and *Rhizoctonia* were among the 20 most abundant genera associated with rice roots, and contain rice-pathogenic species, such as *R. solani* that cause rice sheath blight [54, 55]. One of the two most abundant ASVs assigned to the genus *Fusarium* could be assigned to the species *F. solani*, the second to several species with >99% identity. Finally, two genera detected contain species with described biocontrol effects on rice against blast disease: *Chaetomium* and *Rhizophagus* [9, 56].

The root-associated microbiome from rice growing on a Camargue soil thus included a number of taxa known either to belong to the core rice microbiome, or to be rice pathogens. However, certain taxa generally described as associated with rice were only marginally present, suggesting Camargue conditions (including salinity) select specific microbial communities. Also, the soil used in our experiment was diluted with sterile potting soil, which possibly altered the transplanted microbiome compared to undiluted Camargue soil. However, as Howard et al. (2017) [57] showed that using higher proportions of native soil transfer improves the similarity with the source community (with up to 5% in their study), our use of 20% field soil likely preserved a representative fraction of the Camargue microbiome.

### Alpha-diversity of the rice root-associated microbial communities did not change after plant leaf infection at 7- or 14-days post inoculation

In the present study, neither blast nor brown spot diseases significantly altered the alpha diversity of the bacterial or micro-eukaryote communities in the roots and adherent rhizosphere, either at 7 days post inoculation (dpi) for brown spot or 14 dpi for blast. When comparing diseased plant-associated microbiota with healthy ones, we observe no differences in richness, Pielou’s evenness or phylogenetic diversity across several indicators of alpha diversity.

Many studies have analyzed the impact of disease on microbial communities in different crops. The relationship between microbial diversity and disease development varies between published studies, depending on the compartment studied, the marker used, the host plant, the disease or the sampling time. Differences in the observed richness of bacterial communities in response to plant pathogens have often been observed when focusing on root diseases. Hossain et al. (2021) observed an increase in alpha diversity in response to pea root rot infection in both the root and rhizosphere for bacteria and oomycetes [58]. In contrast, wilt disease in cotton reduced rhizosphere diversity in only one of the two cultivars tested [59]. For fungal alpha diversity, changes due to root disease are less common: fungal alpha diversity did not change with wilt disease in cotton plants [59]. No differences in fungal alpha diversity were found in root samples during root rot in pea, but there was an increase in fungal diversity in the rhizosphere [58].

Similar to our study, when focusing on foliar diseases, it is less common to see broad differences in alpha diversity in the root or rhizosphere-associated microbiomes of healthy and diseased plants. For instance, shotgun metagenomics approach on the rhizosphere microbiome of healthy and corn leaf blight infected maize showed no differences in alpha diversity [60].

Several studies have analyzed the effect of foliar pathogen infection on modifications in the rice rhizosphere microbiome following bacterial leaf blight (BLB) or blast infection [20, 21]. Following BLB foliar infection in rice, H. Jiang et al. (2023) found that the infection reduced bacterial diversity but not fungal diversity in rhizosphere [20]. In a similar study focusing only on changes in rice rhizosphere bacteria over time after BLB infection, alpha diversity index is reduced in diseased plants at early infection (one week) and gradually returns to a stable level over time [19]. Similar to our results, no differences in alpha diversity were found in either rhizosphere or root samples of blast-infected rice sampled from the field compared to healthy-looking samples using bacterial 16 S rRNA gene and fungal ITS2 [21].

As the effect of infection on the alpha diversity index varies across studies depending on different criteria, our results align with those observed in the few studies on rice following foliar infection. Infected plants and their control were grown in the same soil for three weeks before inoculation, and since the infection is foliar while the response we are investigating occurs in the roots, we did not expect drastic changes in the rice root-associated microbiota. Instead, we anticipated more subtle shifts in the abundance of specific taxa.

### Fungal and bacterial communities associated with rice roots respond differently to brown spot or blast disease

In contrast to alpha-diversity, beta diversity indices showed contrasted differences between diseased and healthy plant samples for both foliar diseases. Brown spot infection shifted microeukaryote (*p = 0.045*) but not bacterial (*p = 0.46*) communities in the root-associated microbiota after one week, whereas blast infection altered bacterial (*p = 0.03*) but not fungal (*p = 0.286*) communities after two weeks. Comparing plant microbiome recruitment in both pathosystems is not straightforward since these were not studied at the same time of infection: 7 dpi for brown spot and 14 dpi for blast, since we harvested only after appearance of large symptoms and the two disease differ in their symptom’s appearance and development. We compared the beta-diversity of control samples in both pathosystems (Supplementary Material S5) and observed that a 7 days difference in sampling time induced significant changes in the bacterial and fungal communities (*p-value (PERMANOVA) = 0.001*). This result is consistent with a study that showed that rice root microbiota varies dramatically during the vegetative stages and stabilized only after 8–10 weeks of growth [61]. We thus cannot exclude that this variation over time impact the detection of enriched taxa and that more similar, or dissimilar taxa could have been detected in both pathosystems if these had been sampled at the same time. In addition, the contrasting lifestyles of the two pathogens used in this study must be considered. *Pyricularia oryzae* (formerly *Magnaporthe oryzae*), the fungus responsible for rice blast, is classified as a hemibiotrophic filamentous ascomycete pathogen that initiates infection in living host tissue before switching to necrotrophy [62]. In contrast, *Bipolaris oryzae*, the pathogen responsible for brown spot disease, is predominantly a necrotroph [63]. These differences in trophic strategy are expected to trigger distinct plant immune responses [64] and the production of different defence hormones [65], which could explain the differential shaping of microbial communities in the root and rhizosphere.

On the whole, there doesn’t seem to be a clear-cut rule for determining whether rhizosphere bacterial or fungal communities are more affected as a result of stress on the host plant. For example, in pea rhizosphere-associated microorganisms, bacteria seem to be more affected than fungi [58]. Conversely on chilli, fungal communities were more sensitive to *Fusarium* wilt disease than bacterial communities [66]. In rice, BLB contributes a higher percentage of change in rhizosphere fungal microbial communities than bacterial communities considering a NMDS ranking analyses [20].

A similar study to ours found that rice blast infection did not significantly affect the overall structure of bacterial and fungal endophytes in the roots after seven days [22]. This result is consistent with ours regarding fungal communities but contrasts with our results for the bacterial communities, where we observed significant differences between conditions.

Focusing only on bacterial communities, the shift in root-associated bacterial diversity (β diversity) was significant when exposed to *P. oryzae* but not to *B. oryzae* (Fig. 3B). Differences in microbial communities responses to different pathogens have also been observed in tomato, where foliar infection by *Verticillium dahliae* induced more pronounced shifts in root endosphere diversity than *Fusarium oxysporum* [67].

Once again, although some changes in beta diversity are observed, they remain subtle. Given that all plants initially grew in the same soil, that the same rice variety was used, and that the expected effect is systemic, this is not surprising. The plant’s ability to alter its associated microbiota—and, even more so, to recruit a beneficial microbiota—likely depends on the type of pathogen and the severity of the infection. Additionally, the timeframe used in our study may have been too short to detect significant changes in bacterial and fungal community structures, as suggested by Becker et al. [68].

The observed variations in microbiome response depending on the pathogen and microbial community type (bacterial or fungal) could be attributed to differences in the metabolites exuded by the plant in response to blast and brown spot infections. In a systemic response, and in alignment with the “cry for help” hypothesis, the primary driver of changes in below-ground, root-associated microbiota is the plant’s metabolomic exudation following above-ground infection. Differences in the composition of these exudates between blast and brown spot infections may explain the variations observed in microbiome response. Further studies are needed to investigate how pathogen-specific metabolomic exudation influences microbial community dynamics.

### Recruitment of specific beneficial bacteria upon infection

A wide variety of bacteria are known to enhance plant resistance to disease [69]. We therefore looked for microorganisms enriched in diseased plants as potential biocontrol agents, under the hypothesis that plants recruit microorganisms that help them fight pathogens.

In blast-infected plants, nine genera in the rhizosphere were statistically enriched. Some (e.g., *Acidibacter*, *Ferrovibrio*,* Leptospira*,* Spirochaeta* and *Steroidobacter)* are not typically linked to plants. *Leptospira* includes human pathogens such as *Leptospira ballum*, causal agent of “rice cropping fever” that had already been identified in Camargue in 1954 [70]. In contrast, the genera *Dokdonella*,* Spirochaeta* and *Haliangium* were also found to be enriched in association with blast-infected rice roots. While these genera have already been described for their interactions with plants, the literature only describes the effect of *Haliangium* on plants. This genus synthesises haliangicin, a compound with antifungal properties against numerous phytopathogenic fungi, which has also been linked to growth-promoting effects in several plants, including tobacco, cucumber or vetiver plant [71]. This genus has been found associated with wheat [72, 73] and some species have been isolated from saline environments [74], such as the Camargue soil used in this study. *Lentzea* and *Streptomyces* species are known for their potential as biocontrol agents against *P. oryzae*, and interestingly these were found to be enriched in blast infected plants. Isolates belonging to *Lentzea* as well as *Streptomyces* were tested in vitro and in planta against *P. oryzae* and inhibited the phytopathogenic fungi by >45% [75]. *Streptomyces* species are known to play an important role in agricultural due to their ability to produce various bioactive compounds and their potential biocontrol against phytopathogens including *P. oryzae* [50, 76].

Only two bacterial genera were enriched in brown spot-infected plants (*Herpetosiphon* and *Dokdonella*), which is consistent with the smaller global impact of *B. oryzae* on the global root-associated diversity. These genera have never been described as affecting plants.

Only one bacterial genus was found to be enriched in diseased plants in both pathosystems: *Dokdonella*, a gamma-proteobacterial genus which is not particularly known for its interactions with plants. A closer look at the main ASVs shows that it shares 98.8% identity with *D. soli* and 98.3% with *D. ginsengisoli*, but we couldn’t identify the species solely based on the 16 S rRNA gene fragment. Unfortunately, there is little information on the ecology of *Dokdonella* species, except that they are aerobic heterotrophs with biodegradation capacities in bioreactors and are considered as endogenous denitrifiers [77]. Apart from this genus, there are no similarities in the enriched or depleted bacterial genera between the two diseases. It is not well described whether infection with different foliar pathogens or, more broadly, whether different stresses tend to induce the same recruitment in the plant’s rhizosphere microbiota. However, Flemer et al. aimed to assess the impact of abiotic or biotic stresses on the bacterial composition of the tomato root endosphere [67]. They found out that stress altered the composition of the root endosphere microbiota with a stress-specific shift and a stress-specific enrichment of beneficial bacteria.

Our results identified several bacterial taxa enriched in the roots-associated microbiota of above-ground infected plants, some of which are known for their growth-promoting or biocontrol effects. At this stage, it remains uncertain whether these changes occur randomly or if a particular stress triggers a specific plant response, leading to targeted recruitment. The fact that plants predominantly did not attract the same taxa supports the idea that there is no universal response to fungal foliar infection but rather a pathogen-specific one. While the enriched functional traits may be similar, functional redundancy among bacterial taxa likely masks any common patterns, making them difficult to detect with our current approach. Interestingly, the only common taxon, *Dokdonella*, has not been well-documented in plant interactions. Isolating it through culturomic approaches could provide insights into its potential role in plant health.

### Recruitment of specific fungi upon infection

Following ITS marker analysis, the changes in microeukaryotes associated with roots and inherent soil during foliar infection of rice by either *Bipolaris oryzae* or *Pyricularia oryzae* appear to be pathogen specific. Only the fungus *Cladosporium halotolerans* was enriched in infected plants for both diseases, while *Stachybotrys terrestris* was consistently depleted. *Cladosporium halotolerans* was shown to have plant growth-promoting (PGP) properties on several crops including tomato, bok choy or cabbage [78], and was also able to control the phytopathogenic fungus *Bipolaris spicifera*, confirming its potential as a biocontrol agent [79]. On the other hand, *Stachybotrys terrestris* has not been studied extensively, though a closely related species, *Stachybotrys elegans*, is known to parasitize the phytopathogen *Rhizoctonia solani* [80].

Among the fungi enriched in *Bipolaris*-inoculated plants, one particularly noteworthy species is *Rhizophagus irregularis*, an arbuscular mycorrhizal fungus (AMF) renowned for its plant growth-promoting and biocontrol activities, especially on rice. This AMF species has shown strong compatibility with Nipponbare, the rice cultivar used in this study, demonstrating high root colonization intensity and a reduction in symptoms caused by *Xanthomonas oryzae* pv. *oryzae* [18]. Furthermore, inoculation with *Glomus intraradices*, a closely related AMF, also conferred protection against rice blast on *O. sativa L*. cv. Senia [81]. The increase in *R. irregularis* abundance was however only observed in brown spot-infected plants, not in blast-infected plants. The biocontrol effect of some AMF against brown spot disease has already been tested with AMF on cv. Giza 177, but the AMF species used is not specified [82].

Two fungal species were found to be depleted in brown spot infected plants: *Acremonium persicinum* and *Aureobasidium pullulans. Acremonium persicinum* was screened for the production of an antifungal agent: ASP2397 which inhibits the growth of *Aspergillus fumigatus*, causing invasive pulmonary aspergillosis in human [83]. It has also been used for biological control of tar spot of coconut caused by *Catacauma torrendiella* and *Coccostroma palmicola* in Brazil [84]. The other depleted species, *Aureobasidium pullulans*, is a yeast known for its plant growth-promoting and biocontrol activities, particularly against *Rhizoctonia solani*, causal agent of rice sheath blight [85]. The genus *Rhizoctonia* was abundant in our samples, being the most common fungal genus across all samples but we were not able to identify the species.

In the changes in abundance observed after *P. oryzae* foliar infection, *Fusarium solani*, *Cladorrhinum flexuosum* and *Natantispora unipolaris* were enriched in the diseased plants and *Pseudogymnoascus pannorum* is was depleted. In accordance with our results, *Fusarium solani* is a fungus that is frequently isolated from soil and plant debris, and it produces compounds that can induce systemic resistance in rice against *P. oryzae* [86]. In line with the “cry for help” hypothesis, this suggests that *Fusarium solani* may have been recruited by blast-infected plants for its potential biocontrol activity. *Cladorrhinum flexuosum* was isolated from wheat tissues and positively tested for its biocontrol activities against two wheat pathogens, *Fusarium graminearum* and *Waitea circinate* [87]. No information was found about *Natantispora unipolaris*, also enriched in blast diseased-plants, nor for *Pseudogymnoascus pannorum*, the only fungus depleted exclusively upon blast infection.

The differences in enriched or depleted taxa between pathosystems suggest that the plant’s response to infection—and consequently, the exuded molecules—is specific to the pathogen. Further studies at the transcriptomic and metabolomic levels are needed to validate this hypothesis.

In line with the “cry for help” hypothesis, we identified several fungi enriched in infected plants, some of which are already known for their beneficial potential. For those that are enriched but not yet well-documented, they represent promising candidates for further investigation as potential biocontrol agents. Experimental validation will be required to confirm their role in plant tolerance to above-ground infections. For this study, we selected *R. irregularis* for functional testing due to its availability, but additional candidates should also be evaluated.

### Rhizophagus irregularis has a biocontrol effect against blast disease but not brown spot under greenhouse conditions

In addition to the nutritional benefits, mycorrhization can induce resistance in the plant, allowing it to better withstand certain biotic stresses. This phenomenon, similar to ISR, is called MIR for mycorrhiza-induced resistance [88]. As *R. irregularis* was found to be statistically more abundant in brown spot infected plants compared to the control, the potential biocontrol effect of this AMF on both diseases was also tested. AMF colonisation had no effect on brown spot, but did reduce disease severity in plants infected with *P. oryzae*. This confirms earlier reports of *R. irregularis* enhancing blast tolerance in rice [84], but does not explain its higher abundance in brown spot-infected plants, where no protective effect was observed. This finding underline that the recruitment of a potentially beneficial microbe does not necessarily translate into effective disease suppression. Several factors may account for this discrepancy. First, the AMF inoculum used in our assays (*R. irregularis* DAOM 197198) was a commercial strain that differed from the naturally enriched ASV, sharing 88% identity in the ITS2 region. Still it is the best match of the enriched ASV with a known species, at 88% identity (on 100% coverage), but it might not belong to the same species at this score range. Given that AMF species can exert contrasting effects on their host [18], it would be interesting to test the inoculation of a strain corresponding to the enriched ASV after its isolation from the field. Beyond strain-specificity, other environmental factors such as the soil type and the timing of inoculation may also modulate the ability of AMF to confer protection [89].

## Conclusion

Our findings provide compelling evidence in support of the “cry for help” hypothesis, where plants actively engage their microbial partners to enhance survival and growth, or that of their offspring, when faced with external stressors. Although the changes in rice root-associated microbiota following pathogen infection were not highly contrasted, we observed subtle, yet significant, shifts at the taxonomic level. Notably, certain potentially beneficial bacteria and fungi, such as the genera *Lentzea* and *Streptomyces*, as well as the fungi *Cladosporium halotolerans* and *Rhizophagus irregularis*, showed increased abundance in the rhizosphere of infected plants. These two fungi are of interest because *Rhizophagus irregularis* has been shown to have a biocontrol effect on blast disease. *Cladosporium halotolerans* is an even more interesting candidate as it was found to be enriched in both *Bipolaris oryzae* and *Pyricularia oryzae* infected plants compared to their respective controls. Their role in plant tolerance to above-ground infections will require experimental validation.

Our study also highlights the pathogen-specific enrichment of microbes, with distinct microbial taxa becoming more prevalent depending on whether the plant was infected by *Bipolaris oryzae* or *Pyricularia oryzae*. These findings hint at a complex interaction between the plant’s immune response and microbial recruitment, possibly mediated by the production and accumulation of defensive metabolites. However, the precise mechanisms underlying these microbial shifts remain speculative. To deepen our understanding of how plant health is modulated through microbiota recruitment, future research should focus on identifying the specific metabolites exuded by plants following infection and unravelling the role these exudates play in shaping root-associated microbial communities.

## Supplementary Information

Below is the link to the electronic supplementary material.

Supplementary Material 1

## Data Availability

Sequence data that support the findings of this study have been deposited in the European Nucleotide Archive with the primary accession code PRJEB84129.R scripts for the DADA2 pipeline, rarefaction curves, alpha diversity and metacoder heat trees are freely available on github at https://github.com/lmoulin34/Lea_PyriBlast.
