# Supplementary material for: Variations in plant’s cry for help evidenced by modifications of rice root microbiota induced by blast or brown spot diseases
Source: Environ Microbiome. 2025 Oct 7;20:128. doi: 10.1186/s40793-025-00787-2 (PMC12505648; doi:10.1186/s40793-025-00787-2)

Listing of supplementary material

- **Supplementary Material S1**. Statistically different abundant genera (16S) in *Bipolaris oryzae* (brown spot) inoculated plants compared to control ones.
- **Supplementary Material S2**. Statistically different abundant genera (16S) in *Pyricularia oryzae* (blast) inoculated plants compared to control ones.
- **Supplementary Material S3**. Statistically different abundant species (ITS) in *Bipolaris oryzae* (brown spot) inoculated plants compared to control ones.
- **Supplementary Material S4**. Statistically different abundant species (ITS) in *Pyricularia oryzae* (blast) inoculated plants compared to control ones.
- **Supplementary Material S5.** NMDS of beta-diversity showing the evolution of the global microbial community structure in uninoculated controls at 7 and 14 days post-mock inoculation.

Supplementary Material S1. Statistically differentially abundant genera (16S) in *Bipolaris oryzae* inoculated plants compared to control ones

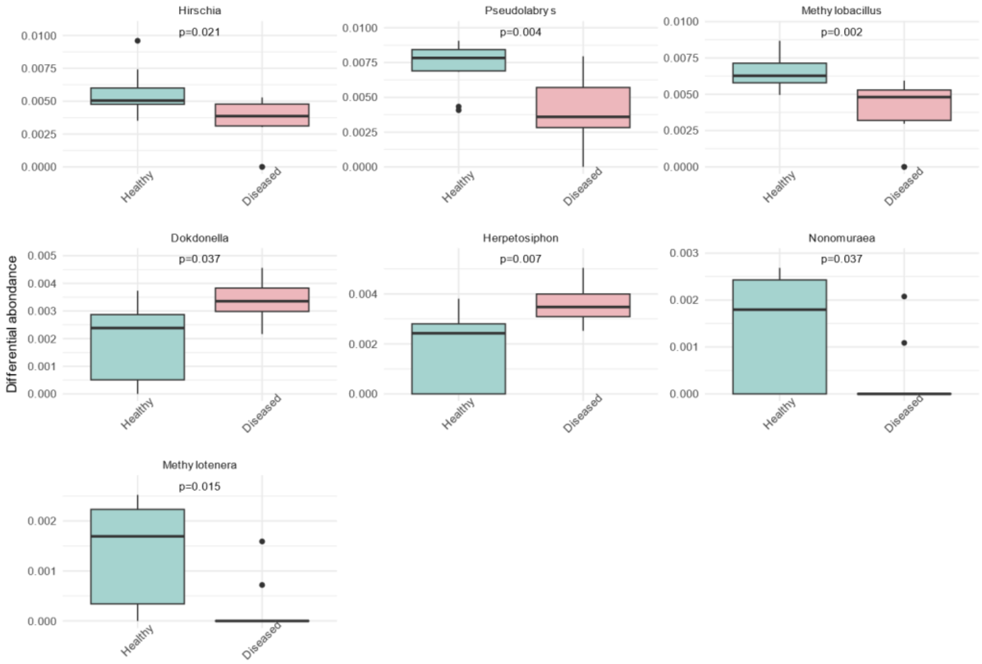

Brown spot (16S)					
**Genus**	**Condition**	**Mean abundance**	**Median abundance**	**Wilcoxon test**	**Augmentation inoculated vs. control (%)**
Dokdonella	Inoculated	0.334319717	0.335315661	0.037	70.4
	Control	0.196168186	0.23835966	*	
Herpetosiphon	Inoculated	0.363499243	0.347534743	0.0071	107.9
	Control	0.174851433	0.242527909	**	
Hirschia	Inoculated	0.339310069	0.386414077	0.037	-39.5
	Control	0.561201955	0.504425786	*	
Methylobacillus	Inoculated	0.388333254	0.480775464	0.0017	-40.2
	Control	0.649077786	0.627489695	**	
Methylotenera	Inoculated	0.023113463	0	0.015	-83.6
	Control	0.140620417	0.169289116	*	
Nonomuraea	Inoculated	0.031626586	0	0.037	-76.7
	Control	0.13583482	0.17957604	*	
Pseudolabrys	Inoculated	0.388437595	0.359553729	0.0036	-46.5
	Control	0.72630838	0.782878757	**	

Supplementary Material S2. Statistically differentially abundant genera (16S) in *Pyricularia oryzae* (blast) inoculated plants compared to control ones

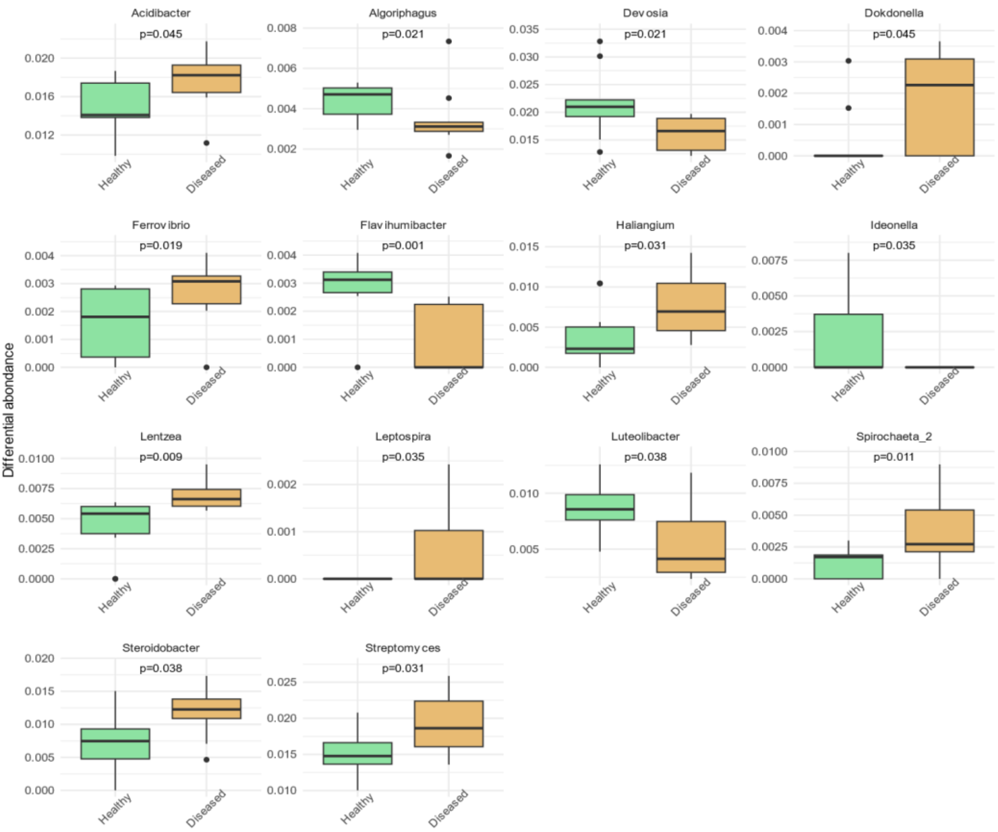

Blast (16S)					
**Genus**	**Condition**	**Mean abundance**	**Median abundance**	**Wilcoxon test**	**Augmentation inoculated vs. control (%)**
Acidibacter	Inoculated	1.764629369	1.822622793	0.043	18.8
	Control	1.48493736	1.407761642	*	
Algoriphagus	Inoculated	0.348678314	0.311902559	0.019	-20.9
	Control	0.440592416	0.471087882	*	
Devosia	Inoculated	1.617492487	1.660403027	0.019	-25.0
	Control	2.157692605	2.098459994	*	
Dokdonella	Inoculated	0.174585745	0.226027829	0.045	282.9
	Control	0.045597896	0	*	
Ferrovibrio	Inoculated	0.273567878	0.308089459	0.019	66.3
	Control	0.164487455	0.180879428	*	
Flavihumibacter	Inoculated	0.092233628	0	0.001	-67.7
	Control	0.285515911	0.312281709	**	
Haliangium	Inoculated	0.740231452	0.69459729	0.031	95.5
	Control	0.378548587	0.230771205	*	
Ideonella	Inoculated	0	0	0.035	-100.0
	Control	0.205136962	0	*	
Lentzea	Inoculated	0.691665402	0.662192158	0.0091	58.6
	Control	0.436087944	0.541452876	**	
Leptospira	Inoculated	0.064644718	0	0.035	-
	Control	0	0	*	
Luteolibacter	Inoculated	0.569890983	0.413620075	0.035	-33.8
	Control	0.860359957	0.856481993	*	
Spirochaeta_2	Inoculated	0.36372119	0.272234271	0.011	186.8
	Control	0.126825355	0.171162177	*	
Steroidobacter	Inoculated	1.182124722	1.224611041	0.035	59.7
	Control	0.740230904	0.745091204	*	
Streptomyces	Inoculated	1.932174475	1.863376517	0.029	28.4
	Control	1.505327492	1.478050602	*	
TM7	Inoculated	0.284355468	0.298662786	0.043	-28.0
	Control	0.394968266	0.365134742	*	

Supplementary Material S3. Statistically differentially abundant species (ITS) in *Bipolaris oryzae* (brown spot) inoculated plants compared to control ones

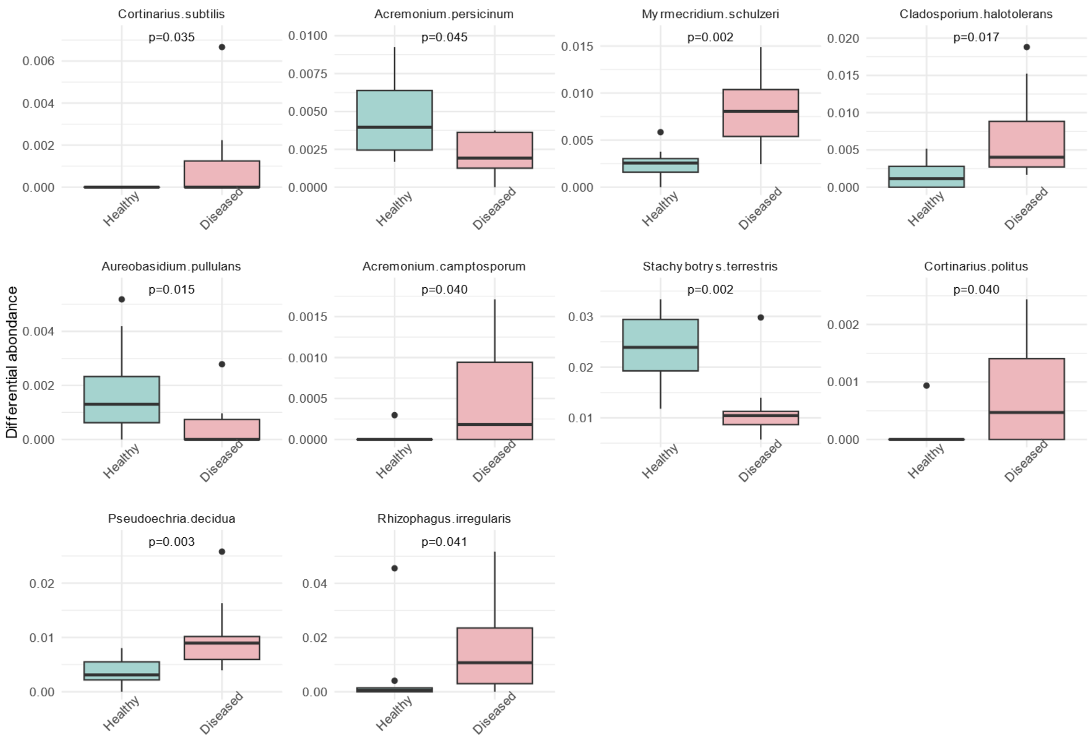

Brown spot (ITS)					
**Species**	**Condition**	**Mean abundance**	**Median abundance**	**Wilcoxon test**	**Augmentation inoculated vs. control (%)**
Acremonium camptosporum	Inoculated	0.055096685	0.018366167	0.04	1755.6
	Control	0.00296914	0	*	
Acremonium persicinum	Inoculated	0.212000069	0.192010065	0.045	-53.4
	Control	0.454878187	0.395933895	*	
Acrobeloides ASV132	Inoculated	0.011497538	0	0.0063	-91.2
	Control	0.130223979	0.143813174	**	
Amphisphaeriales_Is ASV700	Inoculated	0.219202288	0.131736351	0.021	637.5
	Control	0.029724329	0	*	
Aureobasidium pullulans	Inoculated	0.049888104	0	0.015	-72.9
	Control	0.183931906	0.130500667	*	
Cladosporium halotolerans	Inoculated	0.673804019	0.401282482	0.017	292.5
	Control	0.171667198	0.113901555	*	
Cortinarius politus	Inoculated	0.079478427	0.046916881	0.04	748.8
	Control	0.009363768	0	*	
Cortinarius subtilis	Inoculated	0.109880405	0	0.035	-
	Control	0	0	*	
Myrmecridium schulzeri	Inoculated	0.81304521	0.805291335	0.00073	218.5
	Control	0.255292505	0.256090075	***	
Myrothecium ASV699	Inoculated	0.165209879	0.156063691	0.0029	517.1
	Control	0.026770552	0	**	
Oxytrichidae_Is ASV1808	Inoculated	0.158047606	0.090881541	0.015	-
	Control	0	0	*	
Pseudoechria decidua	Inoculated	1.020748895	0.896274759	0.0028	186.1
	Control	0.356822668	0.311337497	**	
Rhizophagus irregularis	Inoculated	1.540441498	1.072760825	0.041	187.4
	Control	0.535901063	0.055710749	*	
Stachybotrys terrestris	Inoculated	1.16132344	1.043757322	0.00073	-50.9
	Control	2.364709374	2.389618734	***	

Supplementary Material S4. Statistically differentially abundant species (ITS) in *Pyricularia oryzae* (blast) inoculated plants compared to control ones

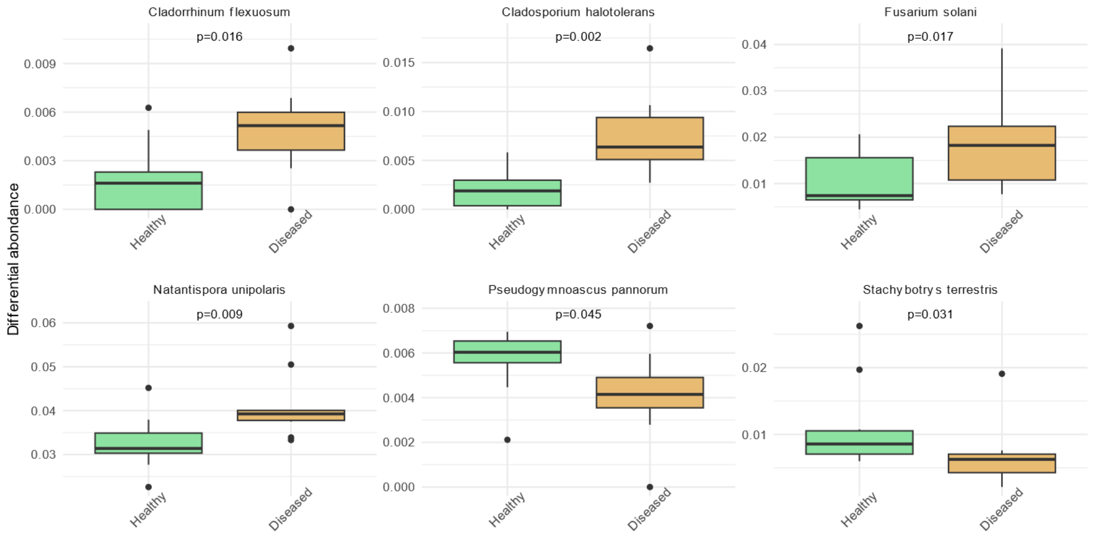

Blast (ITS)					
**Species**	**Condition**	**Mean abundance**	**Median abundance**	**Wilcoxon test**	**Augmentation inoculated vs. control (%)**
Cladorrhinum flexuosum	Inoculated	0.495529621	0.516513647	0.016	162.6
	Control	0.188688267	0.161680202	*	
Cladosporium halotolerans	Inoculated	0.728122127	0.636537208	0.0017	241.6
	Control	0.213127513	0.188698232	**	
Fusarium sp1solani	Inoculated	1.967259648	1.823834279	0.015	91.9
	Control	1.025391561	0.739662601	*	
Natantispora unipolaris	Inoculated	4.11505445	3.924344839	0.0068	26.1
	Control	3.262073683	3.138401726	**	
Pseudogymnoascus pannorum	Inoculated	0.410395703	0.41467129	0.043	-27.6
	Control	0.566857122	0.603382721	*	
Stachybotrys terrestris	Inoculated	0.685277187	0.625717848	0.029	-37.6
	Control	1.097819292	0.855967216	*	

Supplementary Material S5. NMDS of beta-diversity showing the evolution of the global microbial community structure in uninoculated controls at 7 and 14 days post-mock inoculation.

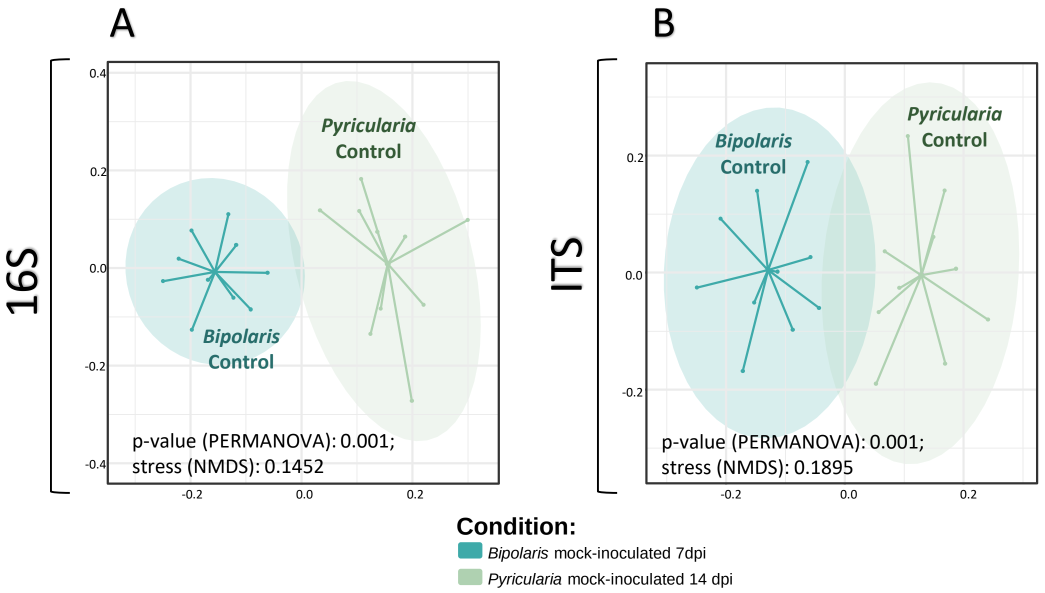

Supplement: Supplementary file 1 — Supplementary Material 1 [file 40793_2025_787_MOESM1_ESM.pdf]
